# Draft Genome Sequence of *Rhizobium* *pusense* Strain NRCPB10^T^ (LMG 25623^T^) Isolated from Rhizosphere Soil of Chickpeas (*Cicer arietinum* L.) Grown in India

**DOI:** 10.1128/genomeA.00282-17

**Published:** 2017-04-27

**Authors:** Jhasketan Badhai, William B. Whitman, Subrata K. Das

**Affiliations:** a Department of Biotechnology, Institute of Life Sciences, Bhubaneswar, India; b Department of Microbiology, University of Georgia, Athens, Georgia, USA

## Abstract

*Rhizobium pusense* strain NRCPB10^T^ was isolated from rhizosphere soil of chickpeas (*Cicer arietinum* L.). Based upon the draft genome sequence, the genome is 5.28 Mb and encodes 5,064 proteins.

## GENOME ANNOUNCEMENT

*Rhizobium pusense* strain NRCPB10^T^ is a nonnodulating, Gram-negative, motile, and rod-shaped bacterium belonging to the class *Alphaproteobacteria*. This bacterium was isolated from rhizosphere soil of chickpeas (*Cicer arietinum* L.) (1). Genome sequencing of this type strain will be helpful in resolving the ambiguity in the phylogeny of the *Rhizobium*/*Agrobacterium* group within the family *Rhizobiaceae* (2).

The draft genome of strain NRCPB10^T^ was generated at the DOE Joint Genome Institute (JGI) using the Illumina HiSeq 2500 platform (3). An Illumina standard shotgun library was constructed and sequenced using the Illumina HiSeq 2500 platform, which generated 8,043,576 reads totaling 1,214.6 Mb. The filtered Illumina reads were assembled using the Velvet (4), wgsim, and Allpaths-LG (5) tools. The final draft assembly contained 29 contigs in 28 scaffolds, totaling 5.28 Mb. The largest and *N*_50_ contigs were 913.6 kb and 405.1 kb, respectively, with a G+C content of 59.2%.

The genome was annotated using the JGI Microbial Genome Annotation Pipeline (6). Genes were identified using the Prodigal (7) and GenePRIMP (8) programs. The tRNAs, rRNAs, and other noncoding RNA genes were identified by searching the genome using the tRNAScan-SE tool (9), rRNA gene models built from SILVA (10), and Infernal (11), respectively. The draft genome contained 5,064 coding sequences, 52 tRNAs, 3 rRNAs, and 10 other RNAs.

### Accession number(s).

This whole-genome shotgun project has been deposited at DBJ/EMBL/GenBank under the accession number FNBB00000000. The version described in this paper is the first version, FNBB01000000.
